# Audiovisual aids in primary healthcare settings’ waiting rooms. A systematic review

**DOI:** 10.1080/13814788.2018.1491964

**Published:** 2018-08-22

**Authors:** Christophe Berkhout, Suzanna Zgorska-Meynard-Moussa, Amy Willefert-Bouche, Jonathan Favre, Lieve Peremans, Paul Van Royen

**Affiliations:** a Department of General Practice/Family Medicine, Lille University, Lille, France;; b Department of Primary and Interdisciplinary Care, University Antwerp, Antwerp, Belgium;; c Department of Nursing and Midwifery, University Antwerp, Mental Health Research Group, Vrije Universiteit Brussel, Brussel, Belgium

**Keywords:** Primary healthcare, health promotion, patient education as topic, waiting room, audiovisual aids

## Abstract

**Background**: Health promotion is part of GPs' commitments. Some waiting rooms have therefore been implemented with audiovisual aids (posters, pamphlets or screens) for health promotion purposes. Few studies have assessed the effect of audiovisual aids in primary care.

**Objectives**: To identify, describe and appraise studies that have investigated the effects of audiovisual aids on health promotion in primary healthcare waiting rooms. To determine which factors influence this impact through literature review.

**Methods**: Systematic review. Two independent researchers using predefined keywords searched databases. Additional publications were extracted from the reference lists of the selected articles. The selection of the articles was performed on the title and abstract, followed by complete reading and assessment. Bias and level of evidence were analysed.

**Results**: A total of 909 articles were collected. Most of them were not in primary care settings. Fourteen peer-reviewed articles fully meeting inclusion criteria were included and analysed. Good quality studies were scarce. Eight of these articles using videos or slideshows on TV screens or tablets indicated effects: three of them were significant on patient knowledge with acceptable evidence and three on health behaviour on surrogate endpoints. Audiovisual aids seem to be used or noticed by patients and can induce conversations with physicians. The relevant factors that might influence these effects (duration of exposure, conception quality, theme, target population and time spent in the waiting room) are insufficiently investigated.

**Conclusion**: Audiovisual aids broadcasting messages using screens (TVs, computers, tablets, and smartphones with Bluetooth^®^ pairing) probably enhance patients’ knowledge. A change in health behaviour remains controversial.

KEY MESSAGES

- Primary care practices make use of their waiting rooms to display many sorts of audiovisual aids (AVAs) to educate or sensitize patients.
- AVAs using screens (TVs, computers, tablets, and smartphones) might improve patients’ knowledge but the size of the effect seems to be small and not necessarily relevant.
- No robust demonstration of a change in health behaviour led by AVAs was found.

## Introduction

Promoting good health has long been part of a GPs’ commitments [1]. Most practices have waiting rooms for patients. In developed countries, 70–85% of patients meet their GP at least once per year [2] and most patients spend time in the waiting room. Most GPs promote good health by hanging posters in the waiting room, offering pamphlets or broadcasting health messages on TV screens, but without a clearly defined strategy [3]. Methods of communication have been changing over time from the usual pamphlet and poster, to TV screens, tablets, computers and programmes associated to smartphones by Bluetooth^®^, and they offer immense potential for health [4]. All media are potentially efficient if used appropriately [5]. At present, there is limited knowledge with regard to how and to what extent these educational aids on health are used in primary healthcare: there are very scarce publications in GPs’ offices, most publications relating to other primary care services, such as family planning centres, or outpatients integrated primary care consulting rooms. Nonetheless, this knowledge is essential to create future educational campaigns [6,7].

This systematic review aimed to look for the best available evidence to inform future primary care practices about the use of audiovisual aids (AVAs) in waiting rooms. Thus, the primary objective was to identify, describe and assess studies exploring the overall impact of AVAs on health promotion within primary health settings and GPs’ waiting rooms. AVAs accounted for posters, booklets/pamphlets or screens (TVs, tablets, computers monitors) broadcasting slides, videos or computer programs. The secondary objectives were: (i) to identify and assess the factors that contributed to these effects; (ii) to assess whether the effects induced by posters and pamphlets differed from those induced by screen-based aids.

## Methods

### Study design

For this systematic review of the effects of AVAs (posters, pamphlets and screens) on health promotion in waiting rooms of GPs or other primary healthcare services, we followed the Cochrane handbook [8] and included all observational and intervention studies with summaries published from 2004 to 2017.

### Search strategy

The studies had to occur outside hospitals and directed to primary healthcare, defined as general practice and paediatric surgeries (offices), sexually transmitted diseases (STD), family planning and mother and child clinics to be eligible. Studies could be observational, clinical trials or phenomenological approaches. AVAs could be posters, pamphlets, videos on television screens, computers or tablets, education programmes displayed by Bluetooth^®^ association on smartphones, or computer-based education software. The aids had to be visible or available in waiting rooms.

### Selection of studies

The main outcome was the description of one or more AVAs on health promotion in GPs’ or other primary healthcare services waiting rooms, and the assessment of the aid’s effect. The secondary outcome was to identify and assess the factors that contributed to this effect.

A search equation using MeSH keywords was tested before exploring the databases: (Primary health care OR family practice OR general practice NOT hospitals) AND (patient education as topic OR health promotion) AND (audiovisual aids OR advertising as topic OR pamphlets OR posters).

**Figure 1.**
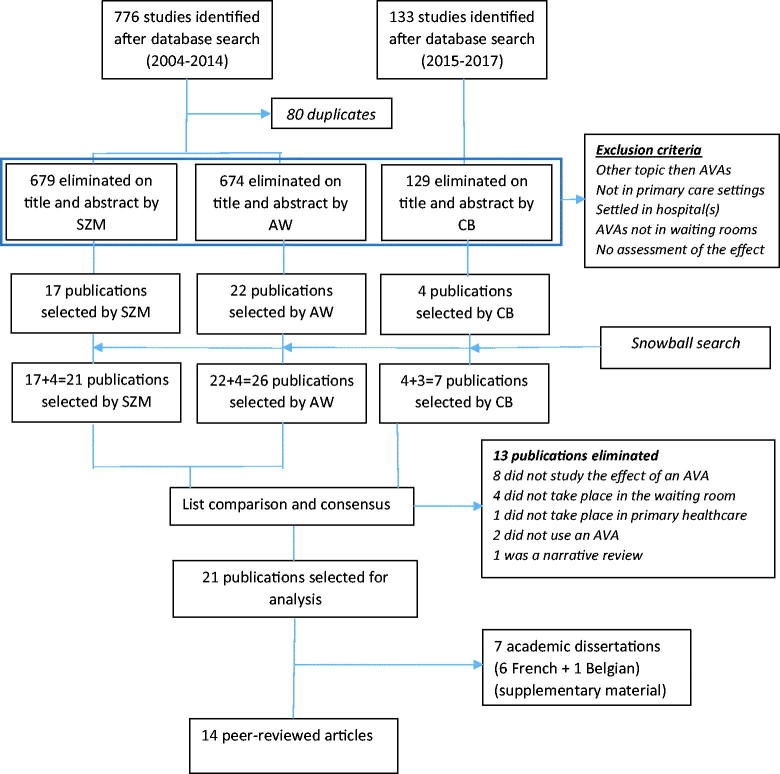
Flow chart of manuscripts selection.

Medline, Web of Science, Cochrane Library, Scopus, Google Scholar, and SUDOC (Central French Universities Documentation Service) were searched by two independent investigators from 1 January 2004 to 31 December 2014 and a complementary search from 1 January 2015 to 31 December 2017, with language availability restricted to English and French. Among the collected publications, duplicates were eliminated, and then titles and abstracts were read. Articles were excluded if they occurred in hospitals or elsewhere than in primary care settings, when they dealt with topics other than AVAs or with AVAs elsewhere than in waiting rooms or not assessing the effect of the AVAs. The references of eligible publications were analysed and relevant publications were retrieved. At this stage, the agreement between the lists was evaluated by Cohen's Kappa. Consensus finalized the list of publications included for analysis after the articles had been entirely read (Figure 1).

### Quality assessment

The quality of the articles was assessed by one researcher for each selection through the CONSORT table for intervention studies [9], and the STROBE for observational studies [10]. Biases and the quality of evidence were evaluated via the GRADE criteria and the Cochrane handbook tool [8,11]. The risk of bias was not a selection criterion. Peer-reviewed articles were separated from non-peer-reviewed (academic dissertations), presented as a supplement.

### Data analysis

One researcher extracted the relevant data for analysis purposes. All primary or secondary outcome measures were taken into account, and any limitation of the studies was classified as a bias. Because of the heterogeneity of the studies, the lack of a common outcome, and the low level of evidence in most studies, no meta-analysis could be undertaken (Table 1, last column).

**Table 1.** Principal characteristics of the peer-reviewed articles included in the current review.

Study ID	Practice speciality	Audiovisual aid(s) used in the studies	Topic or general purpose of the study	Aid display	Design, data source	Number of patients	GRADE assessment	Outcome(s) studied	Main results
1-Ashe [13]	Paediatrics	Poster	Antibiotic prescriptions for children with respiratory illnesses	1 month	Historical comparison in doctors’ prescriptions	720	Very low	PP	PP: not SS
2-Assathiany [14]	Paediatrics	None, poster, pamphlet, (TV, videos)	Every educational issue regarding children	Unknown	Survey parents + physicians	1830	Very low	K, U, D, PP	K, U, and D: theme dependant, no significance calculation
3-Stephens [15]	PHC	Poster	Occurrence of patient–physician weight loss conversations	5 days	Quasi-experimental	668	Low	D	D: not SS
4-Eubelen [16]	GP	Videotape recording	Tetanus booster vaccination prescription by the GP	6 months	Quasi-experimental. Local pharmacists	20,109	Moderate/low	HB	HB: increase vaccination prescriptions (0.79% vs 0.39%, *P* < 0.0001)
5-Frosch [17]	PHC	Videotape recording vs pamphlet	Cancer screening: to enhance autonomous decision-making	Once	Quasi-experimental, separate room, recruited by staff	207	Moderate/low	D, HB, K	D and HB: not SS; K: increase with video vs pamphlet (no significance calculation)
6-Goodman [18]	PCC	Videotape recording	Influenza vaccination of pregnant women	3 months	RCT, separate rooms, interview by phone	105	Moderate	HB, K	HB: not SS (*P* = 0.70); K increased protection by vaccination (mother difference = 0.49, *P* = 0.003; baby difference = 0.59, *P* = 0.001)
7-Myint-U [19]	STD	Videotape recording, poster	Reduce the acquisition of new infections	4 weeks	Survey + staff observation	1096	Low	U	U: interest of the video: 49–85% watched
8-Warner [20]	STD	Videotape recording, poster	Reduce the acquisition of new infections	11 × 4 weeks	RCT; 3 centres, clinical records + local registries	38,635	Moderate	HB, U, K	HB: decrease of encounters for new infection: (HR = 0.91; 95%CI: 0.84–0.99) K, U: increased perception of STD risk
9-Birukila [21]	PHC	Video clip shareable on smartphones via Bluetooth®	Polio vaccination uptake in low literacy communities	Unknown	Staff observation	Unknown	Very low	HB	HB: doubled polio vaccine uptake (no significance calculation)
10-Carlfjord [22]	PHC	Computer	Prevention of alcohol use disorders and sedentariness	1 year	Survey, lifestyle test questionnaire	3027	Low	U	U: interest for 5.7% respondents amongst patients
11-Gerber [23]	GP	Computer	diabetes education targeting individuals with low health literacy levels	1 year	RCT, 5 centres, lifestyle test questionnaire	183	Moderate	HB, K	HB: HbA1c: not SS (–0.2 ± 2.0); K: not SS (0.32 ± 2); U: increase of perceived self-efficacy (1.51 ± 1.5)
12-Gilliam [24]	FPC	Tablet	Promotion of long-acting reversible contraceptive methods in deprived women	Unknown	RCT, tablet vs usual care, questionnaire, review of chart	52 (per-protocol)	Moderate	D, K, U,	D: more discussion with physician (7.1 vs 32.1%, *P* = 0.02), K: increase for LARCM and U: interest of the message
13-Larsson [25]	PHC	Computer (digital signage technology)	Social marketing message for increasing radon program participation	3 years	Quasi-experimental, 3 centres, crossover: intervention vs usual	99	Low	HB	HB: increase of radon test kit purchase (*t* = 2.69; 95%CI: 1.20–8.47; *P* = 0.01) and nutrition programme (χ² = 3.13; *P* = 0.077).
14-Pawar [26]	OPC	Pamphlets, vdeotape recording, ‘special events’	Educational information on healthy eating, exercising, and smoking cessation	1 year	Survey, staff assisted	79	Very low	HB, K	HB: 13% quit smoking (no significance calculation); K: not SS

GP: general practice; FPC: family planning clinic; LARCM: long-acting reversible contraceptive methods; OPC: outpatients psychiatric clinic; PCC: prenatal care centre; PHC: other primary healthcare service; STD: sexually transmitted disease; SS: statistically significant; RCT: randomized controlled trial; D: discussion with the physician; HB: health behaviour change; K: knowledge improvement; PP: physician prescription change; U: usefulness or interest of the message; HbA1c: glycated haemoglobin.

Recommendations from the PRISMA statement were used for the presentation of this review [12] and the PRISMA checklist is annexed as supplementary material. Peer-reviewed articles were separated from academic dissertations, and papers reporting on posters and booklets/pamphlets were analysed separately from those reporting on other AVAs. A systematic narrative synthesis was established, information was presented in text form and tables to summarize the analysed articles.

## Results

### Study selection

The study selection flow chart is displayed in Figure 1. The search by keywords through databases yielded 909 publications. Eighty duplicates were removed. The vast majority of articles were settled in hospitals or locations other than primary care settings, were assessing education programmes not basing on AVAs, had AVAs displayed elsewhere than in waiting-rooms (mostly pamphlets handed out by the doctor himself, as support of a short intervention), or did not assess the effects of the aids (for instance carried out a quality assessment of pamphlets or booklets with regard to the level of literacy of targeted patients). The title and abstract selection yielded 21 and 26 articles respectively, and the second search five supplementary. After mutual consensus, 21 publications fully responding to the eligibility criteria were included for analysis, consisting in 14 peer-reviewed articles and seven academic dissertations. This paper only reports on the peer reviewed articles, academic dissertations being presented as a supplement.

### Study characteristics

Table 1 reports the characteristics of 14 peer-reviewed articles [13–26], including one in French [14] and the rest in English [13,15–26].

#### The setting

(Table 1, column 2: ‘Practice speciality’) was diverse primary healthcare services, only two studies being explicitly run in general practice [16,23]. In several cases, the typology of primary healthcare was not mentioned (‘routine PHC’) [15,22,25], or seemed orientated towards preventive care [17,21]. In six cases, settings were orientated towards mother and child services [13,14,18], family planning, and sexually transmitted diseases [19,20,24]. One study was conducted in a community psychiatric clinic [26].

#### The design

(Table 1, column 6, ‘Design, data source’) consisted of four open-label randomized controlled trials [18,20,23,24], and four quasi-experimental studies [15–17,25]. The remaining studies were one historical (before vs after intervention) trial [13], and five cross-section surveys or other observational studies [14,19,21,22,26]. Two articles were probably related to the same study, one publishing the result of a single centre in a multi-centre trial [20]: for this reason, these two articles are not to be considered as independent studies [19].

#### The quality

Table 1 (column 9, ‘GRADE assessment’) of most articles was mediocre. Not a single study was international and, excepted Warner [20], Gerber, and Larsson [23,25], they were monocentric with sampling bias. The number of subjects to be included was not calculated in the methodology of most observational or quasi-experimental studies and subsequently, the statistical power was unknown. The results were often reported without confidence intervals. The intervention about the settings’ usual environment was not detailed in the methodology. The absence of external validity prevents all findings being extrapolated to an international population and the majority could not reasonably be extrapolated beyond the participants.

Used AVAs are summarized in Table 1, column 3. Audiovisual aid(s) used in the studies: three studies were based on posters and/or pamphlets dispatched in waiting rooms [13–15], one describing a complementary use of videos on TV screens in two waiting rooms out of 87, without separate analysis [14]. Five were reporting on video interventions on TV screens [16–20], one comparing video as intervention vs pamphlets as controls [17]. One study reported on videos dispatched on smartphones via a Bluetooth^®^ pairing [21]. Four studies described computer programs, two using lifestyle tests and printed tailored advices regarding alcohol use and physical activity or diabetes [22,23], and one a tablet program promoting long-acting reversible contraceptive methods [24]. The fourth computer program used digital signage technology to broadcast a social marketing message [25]. Finally, one study used pamphlets, DVD videos and ‘special events’ (like poster competitions) [26].

### Risk of bias and limits within studies

There were numerous biases and limits in most of the studies. All the articles that targeted the behavioural change in patients used surrogate endpoints (as reduction of incident STD records rather than a change in sexual behaviour [20]) and some investigated the doctor’s behaviour [13,16,26]. In one case, the study had been designed for a different purpose than the impact of some AVAs so the validity of the findings concerning the effect of the AVA was diminished [17]. Some studies sought to demonstrate the effects in a specific subgroup of patients but as the study population included all patients, the effects found could not be attributed to the targeted population [17,23]. The lack of description in the methodology with regard to (i) location of the AVAs, (ii) level of exposure, and (iii) volume, prevents reproduction of the precise design of the study intervention. One study correlated the waiting time to the level of the effect but the evidence was insufficient [20]. In some studies, the aids were not only present in the waiting room but also in other locations [13,15,17,19,20,22], and it was impossible to know to what extent the effect was due to the aids in the waiting-room. Mostly the studies were conducted on small-sized populations and results did not reach statistical significance, some suggesting a possible trend.

### Results of individual studies

Table 1 (last two columns, ‘outcome(s) studied’ and ‘main results’) summarizes the results. The outcomes described in the articles were organized into sub-groups to facilitate comparison between studies, the first cited outcome being the primary one.

Considering the outcomes searched for in the different studies without accounting whether the outcome was the primary one or the level of evidence, health behaviour (HB) was increased in five studies using screens [16,20,21,25,26] out of eight. All of the articles focusing on HB used a surrogate endpoint, the closest to a clinical effect being the delivery of tetanus vaccine units in community pharmacies on medical prescription (thus a change of behaviour in GPs) [16] or the important upgrade of polio vaccination without formal assessment of the causal link [21]. Knowledge (K) was increased in four studies using screens [17,18,24,26]. In one study using a computer program, knowledge was negative [23]. All the studies using posters and pamphlets solely [13–15] had negative results.

Further data analysis was descriptive, sometimes bivariate but not adjusted on confusing factors. The optimal amount of time for AVAs to be displayed ranging from one to six months is not supported by evidence [20]. Messages aiming at the general population seem to have more effect than those targeting a specific group of patients. GPs’ patients and those consulting STD clinics seem the most receptive to the messages. The presence of other AVAs on different topics does not seem to affect the effect of the assessed AVAs negatively. The use of one or various aids about one or several subjects does not impair the efficiency of the aids. However, the presence of other AVAs is generally not mentioned. When considering posters/pamphlets and videotape recordings/slideshows and computer sessions, videotape recordings/slideshows appear to be more effective on HB change and knowledge. Video clips sharable on smartphones via Bluetooth^®^ were associated with an important increase in polio immunization but the link of causality is weak and the level of evidence is low. The most efficient AVAs were those created by doctors/primary health teams for their waiting room, elaborated alone or together with input from reference centres (health authority agencies).

### Additional analysis

Computer programs have not demonstrated any attested efficacy [22,23], when not considering social marketing with digital signage technology [25]. Computers were implemented in clinics directed at low literacy populations. The authors acknowledge they overestimated the skills of these populations, not trained in the use of computers. The failure to prove any effect is probably due to a failure of patients to use computers.

## Discussion

### Main findings

This review noted a considerable heterogeneity in different AVAs used in primary care waiting rooms, in their purpose to educate or sensitize patients and in the design used to assess their efficacy. Most of the authors’ attitudes were favouring the usefulness of these AVAs, indicating that their implementation in waiting rooms should follow specific rules to ensure their efficiency. However, the formal assessment of AVA is sustained by scarce literature. Posters or pamphlets exposed in waiting rooms did not demonstrate any effect. Videotape recordings/slideshows on TV screens and tablets appear effective on the increase of knowledge with an acceptable level of evidence but evidence remains insufficient to sturdy demonstrate they induce a change in health behaviour. Computer programs have not demonstrated their efficacy, but they were implemented in low literacy populations, not trained in the use of computers.

### Strengths and limitations

This review has limitations. For the first search (2004–2014), there was no double-blinded analysis of the selected articles. However, the selection of the analysed articles was completed by two blinded researchers that led to a high level of agreement. The analysis of the selected articles was conducted according to the Cochrane handbook and it used the GRADE rating scale to evaluate the strength of evidence [8,11]. As the first search was ending in 2014, an update was necessary for this article. Only one researcher carried out this update over three years (2015–2017). The method for selection was almost the same: same keywords to search the databases, same review of references, same selection process. As we had noticed in the first search that no articles were found in the Cochrane Library, and that the articles retrieved from Scopus, Google Scholar and in the Web of Science had led to duplicates with Medline, only Medline was searched. The search in the SUDOC had only led to the selection of French academic redactions, not peer-reviewed, and with a very low level of evidence: it was skipped from the second search process, looking only for peer-reviewed articles. As the selection process over 10 years in the first search had led to a high level of agreement, we presume that a search and selection by only one researcher over three years would not have led to a substantial selection bias.

Another risk of selection bias was the choice of only two languages (English and French) to search publications. The search in other languages or in other sources might not have produced a more extensive range of studied effects or evidence.

Regarding the limitations related to the articles retrieved for this review, the lack of detailed description about the intervention in the methods section makes it difficult to reproduce and to compare. Most studies were biased and surrogate endpoints were often used. The presence of AVAs in waiting rooms is one way among others to educate and inform patients. It was a difficult point of our review, as in some studies other insufficiently described means were implemented and it was not possible to support the part of AVAs in the outcome. Many articles excluded in this review were reporting on passing pamphlets/booklets out during encounters, to reinforce the message of a short intervention.

### Comparison with existing literature

Regarding the setting, the importance of establishing a strategy for health promotion is often present in the literature. Aids should be adapted to a wide population amongst the people likely to be present to address the maximum number of patients. This notion is compatible with that of prevention, which targets the general population [27].

Regarding the relevant factors that might influence the effects of AVAs, doctors ought to participate in conceiving and designing audiovisual supports. On the one hand, this relies on the fact that doctors know their patients best [28]. On the other hand, the GPs’ personal involvement affects their motivation to apply the message displayed by the AVA(s): a probable change in their care behaviour during their encounters with patients creates a bias in the study results (increased Hawthorne effect, not taken into account as confusion factor) [29]. The guidance and advice of public health agencies improve AVAs quality and validity [30]. Not all the promoted topics were equally efficient, probably because of the heterogeneity of the patients’ interest. This is also described in the literature [30]. It is for this reason that some institutions suggested that patients participate in the creation of campaigns [28,31].

The duration the aid is displayed must not be too short or extended to give enough time to patients to notice it and avoiding it getting banal and ignored. However, the optimal duration is not found in the literature. Regarding seasonal interest (seasonal influenza, sexual behaviour), the topic of the study must be adapted to the period or season [32]. Considering the effect of AVAs related to waiting time, evidence from the studied publications is insufficient and more research is necessary to find out if both are associated and if an optimal waiting time exists.

Evaluation of the inter-influence of other AVAs on the studied effect was impossible in the analysed publications so we were unable to determine if a combination of aids is more efficient than a single one. Nonetheless, only slideshows or videos broadcasted on screens were found associated with an increase in knowledge or some change in HB. The cost of creating AVAs during the implementation of campaigns for health promotion in waiting rooms is a constraint raised by the authors but not analysed. The location of support is also a neglected factor in the publications, despite its relevance to the development of support.

Except for three studies where significant results are prone to important biases [18,24,25], the need for more than 10,000 participants in the trials with the best evidence to reach statistical significance indicates a low effect size requiring large studies. Even though the outcome is statistically significant, the effect is small and might have no clinical relevance. Our study team recently published a multicentre cluster randomized controlled trial on more the 10,000 targeted patients using posters and pamphlets in GPs’ waiting rooms that could not demonstrate any increase of seasonal influenza vaccination, acknowledging the lack of efficiency of posters and pamphlets [33] .

## Conclusion

This systematic review leads the authors to believe that videos or slideshows broadcast on screens (TVs, tablets or smartphones using a Bluetooth^®^ pairing) in primary care waiting rooms may contribute to improving patient’s knowledge, but the effect might be small, not necessarily relevant, and prone to a Hawthorne effect in the healthcare team. A change in HB on clinical (not surrogate) endpoints has yet to be sturdily demonstrated. Robust controlled trials on large populations, with a clear design and method are required to prove changes in patients’ behaviour resulting from media campaigns developed in waiting rooms.

## Supplementary Material

PRISMA 2009 Checklist

Supplementary Results and discussion
