# Supplementary material for: Audiovisual aids in primary healthcare settings’ waiting rooms. A systematic review
Source: Eur J Gen Pract. 2018 Aug 22;24(1):202–10. doi: 10.1080/13814788.2018.1491964 (PMC6104610; doi:10.1080/13814788.2018.1491964)
Supplement: Supplementary Results and discussion [file IGEN_A_1491964_SM2663.docx]

**Supplementary Results and discussion: French and Belgian academic dissertations**

The investigation of the SUDOC led to the selection of six French academic dissertations [1–6] (Table 1 suppl), and Google scholar to a Belgian one[7]. All were written by primary care registrars in French, with poor level of evidence, but acknowledging the interest of young GPs for this topic. None of the French dissertations had led to an article, but one mainly qualitative article cited in the introduction of the main article [8], might be related to a redaction [4] reporting on a mixed method study (qualitative and cross-sectional). Regarding Eubelen, the article and the academic redaction are based on the same intervention and the same population, the methods being detailed in the academic redaction [7] than in the article [9], leading to a better quality assessment. Nonetheless, the number of patients is surprisingly not the same (40 684 in the redaction vs. 20 109 in the article).

Increase of effects were only demonstrated for slideshows. Knowledge was only found increased in Dupuis [2] versus poster/pamphlets (+11.5%), without power or significance calculation. Health behaviour was found significant in Eubelen [7] on the prescription of vaccine units by the GP. These vaccine units were delivered in community pharmacies. Injections were not retrieved.

The quality of these redactions is very low, excepted Dupuis and Eubelen, and their content has not been rectified after defence, considering the remarks of the jury. No reasonable conclusion can be founded on these writings.

**References**

[1] Boulard E. [Do posters in waiting-rooms influence consultation claims ?] [Academic redaction]. [France]: Université Jean Monnet (Saint-Étienne). Faculté de médecine Jacques Lisfranc; 2013.

[2] Dupuis A-C, Mouterde-Lefebvre H. [Designing and assessing of a new education modality in primary health: the monothematic and multi-vector education campaign in waiting-rooms (association of posters, pamphlets, slideshow and video). How can primary care practitioners implement such an education campaign (theoretical grounding and practical aspects)? What is the efficacy of this new education modality related to the used communication vectors?] [Academic redaction]. [France]: Université de Rouen; 2010.

[3] Guiho H. [Patient education by the general practitioner: part of a visual medium to broadcast messages in waiting rooms] [Thèse d’exercice]. [France]: Université européenne de Bretagne; 2011.

[4] Idris H. [Part of the general practitioner’s waiting room in health education from general practitioners’ and patients’ perspectives in the Somme department] [Academic redaction]. [France]: Université de Picardie; 2009.

[5] Lagorce S. [Favouring consultation claims about urinary incontinence in women aged from 18 to 80 years by a visual information in waiting-rooms: feasibility study amongst general practitioners and patients in the Bergerac district] [Academic redaction]. [1970-2013, France]: Université de Bordeaux II; 2013.

[6] Rolland M-A. [Comparative study of three education strategies in general practice waiting-rooms regarding vaccination] [Academic redaction]. [France]: Université de Picardie; 2013.

[7] Eubelen C. [Audio-visual message in the general practitioner’s waiting-room: a health education tool] [Academic redaction]. [Liège (B)]: Université de Liège; 2009.

[8] Gignon M, Idris H, Manaouil C, et al. The waiting room: vector for health education? The general practitioner’s point of view. BMC Res. Notes. 2012;5:511.

[9] Eubelen C, Brendel F, Belche J-L, et al. Effect of an audiovisual message for tetanus booster vaccination broadcast in the waiting room. BMC Fam. Pract. 2011;12:104.

***Table 1 (suppl) Principal characteristics of the French and Belgian academic redactions included in the current review.***

| ***Study ID*** | ***Practice speciality*** | ***Audio-visual aid(s)*** | Topic | ***Aid display*** | ***Design, data source*** | ***Number of patients*** | ***GRADE Assessment*** | ***Outcome(s) studied*** | ***Main results*** |
| --- | --- | --- | --- | --- | --- | --- | --- | --- | --- |
| ***1-Boulard, 2013 [1]*** | *GP* | *Poster* | *Searching an association between posters displayed in the waiting-room and consultation claims* | unknown | *Observation during Consultation (nested study of ECOGEN)* | *10 052* | *Low* | *D* | *Discussion increased about memory disorders, dementia ant HPV vaccination (no significance calculation)* |
| ***2-Dupuis, 2010 [2]*** | *GP* | *Posters, pamphlets, slides* | *Comparison of slides vs. posters + pamphlets vs. all 3. About sleep disorders* | unknown | *Historical comparison (before vs. After). Questionnaire* | *1 014* | *Low/Very Low* | *K* | *The knowledge of the patients was increased by 19% with the slideshow. The difference with the posters + pamphlets was 11.5%. (no significance calculation)* |
| ***3-Guiho, 2011 [3]*** | *GP* | *Slideshow* | *Interest in patients of diverse audio-visual aids in the waiting room* | *6-8 weeks* | *Observation + survey* | *510* | *Very Low* | *U, D* | *Majority used audio-visual aid* |
| ***4-Idris, 2009 [4]*** | *GP* | *Poster* | *Attitudes of GPs regarding audio-visual aids in the waiting room* | *6 months* | *Structured interviews, survey* | *85* | *Very Low* | *K, HB* | *5-7.5% cite half posters present* |
| ***5-Lagorce, 2013 [5]*** | *GP* | *Poster, pamphlets* | *Promote requests regarding urinary incontinence in women* | *2 weeks* | *Questionnaire survey* | *187* | *Very Low* | *D* | *No increase of complaint for urinary incontinence in affected women during consultation (rate of complaints unchanged: 68.4%):* |
| ***6-Rolland, 2013 [6]*** | *GP* | *Game, pamphlets* | *Comparison of a game, pamphlets* and usual care *to promote vaccination in children* | *2 months* | *Questionnaire survey* | *212* | *Very Low* | *U, D* | *U and D : no SS difference in between groups and with usual care.* |
| ***7-Eubelen, 2009 [7]*** | *GP* | *Video slideshow* | *Tetanus booster vaccination uptake* | *2x6 months* | *Quasi experimental study. GP records + pharmacists (claim database)* | *40 684* | *Moderate/Low* | *HB, PP* | *Increase of vaccine units prescriptions by the GP, delivered in community pharmacies* |

***Keys: GP****=General Practice; ECOGEN=étude des Eléments de la COnsultation en médecine GENérale.* ***SS****=Statistically significant.*

***D****: Discussion with the physician,* ***HB****: Health behaviour change,* ***K****: Knowledge improvement,* ***PP****: Physician prescription change,* ***U****: Usefulness or interest of the message*
